# Effect of short-term high fat diet on resistin levels and expression of autophagy-related genes in the cartilage of male rats

**DOI:** 10.1038/s41598-022-19481-1

**Published:** 2022-09-12

**Authors:** Hebatallah Mohammed Aboudeya, Sara A. Shaker, Mohammed Salama

**Affiliations:** 1 Human Physiology Department, Medical Research Institute, Alexandria University, 165, Horreya Avenue, Hadara, Alexandria, Egypt; 2 Biochemistry Department, Medical Research Institute, Alexandria University, Alexandria, Egypt; 3 Histochemistry and Cell Biology Department, Medical Research Institute, Alexandria University, Alexandria, Egypt

**Keywords:** Physiology, Diseases, Pathogenesis

## Abstract

Obesity is a significant risk factor for the development of knee osteoarthritis (KOA). However, the precise molecular mechanisms linking obesity to OA remain unclear. In the present study, we investigated the effect of short-term high-fat diet (HFD) on the development of OA and the possible role of the adipokine resistin and autophagy-related genes in mediating this effect. Thirty adult male Wistar rats were equally divided into 2 groups: control and obese groups. Body mass index (BMI), levels of lipid profile, glucose, insulin and HOMA-IR index were significantly higher in the obese group compared with control. Our results revealed significantly higher serum and cartilage resistin levels with a significant increase in the mRNA expressions of toll-like receptor 4 (TLR4), matrix metalloproteinase-9 (MMP-9) and interleukin-1β (IL-1β) as well as protein levels of IL-1β, matrix metalloproteinase-13 (MMP-13), ADAMTS 5 (aggrecanase-2) and caspase-3 in the cartilage of obese rats. The HFD induced a significant upregulation of autophagy related 5 (ATG5), beclin-1 and light chain 3 (LC3) mRNA expressions and a significant downregulation of mammalian target of rapamycin (mTOR) expression in cartilage. The protein levels of cartilage ATG5 were also significantly elevated in HFD-fed group. In conclusion, we suggested that increased levels of resistin and expression of autophagy-related genes may contribute in part, to OA development in HFD-fed rats. This provides a novel insight into the early molecular changes in the cartilage associated with obesity.

## Introduction

The prevalence of obesity has increased dramatically in recent decades and it is now one of the most serious health problems, with more than 1.9 billion adults are overweight and over 650 million of them are obese^1^. Obesity is recognized as a major risk factor for several diseases, including osteoarthritis (OA)^2^. OA is the most common degenerative joint disease, characterized by degradation of articular cartilage, synovial inflammation, and structural changes in bone including the formation of osteophytes and subchondral bone sclerosis^3^.

Obesity-related OA has been described as a separate “metabolic OA” phenotype, representing approximately 60% of OA population^4^. Despite the well-established link between obesity and OA, the impact of a short-term HFD on the development of OA and the underlying molecular mechanisms remain unclear.

In recent years, it has been suggested that increased adiposity in metabolic OA is associated with increased concentrations of adipokines that promote a state of chronic, low-grade inflammation contributing to joint damage^5^. Resistin is a 12 kDa cysteine-rich polypeptide hormone secreted by adipose tissue in macrophages and adipocytes in humans and mice, inducing inflammation and insulin resistance^6^. The adipokine resistin is highly expressed in serum and synovial joints of patients with OA^7^. In human articular cartilages, resistin induced the expression of matrix degrading enzymes and proinflammatory cytokines^7–9^. In addition, resistin has been shown to increase vascular adhesion molecule-1 expression on synovial fibroblasts in OA, whereas the decrease of resistin activity prevented anterior cruciate ligament transection-induced damage to OA rat cartilage^10^.

Autophagy is a crucial homeostatic mechanism that regulates cell metabolism and removes damaged macromolecules and organelles to maintain cellular equilibrium^11^. It may be separated into the following phases artificially: autophagosome induction and nucleation, autophagosome elongation, autophagosome maturation and degradation^12^. Autophagy process is regulated by spatiotemporal coordinated recruitment of autophagy-related proteins (ATG) (the “core machinery”) and accessory proteins^13^. Numerous recent studies demonstrated the importance of autophagy in normal cartilage maintenance and suggested that dysregulation of autophagy is closely associated with the pathogenesis of OA^14,15^. Autophagy was shown to be increased in OA-affected cartilage, according to several studies^16^; however, others found that autophagy was significantly reduced^17^.

In the present study, we aimed to investigate the effect of short-term high-fat diet on the development of OA in HFD-fed male rats and the possible role of the adipokine resistin and autophagy-related genes in mediating this effect.

## Materials and methods

### Ethical statement

The current protocol was approved by Alexandria University- Institutional Animal Care and Use Committee (AlexU-IACUC, Approval number: AU 0122232233). All experiments fulfill the guidelines of the National Institutes of Health guide for the care and use of Laboratory animals (NIH Publications No. 8023, revised 1978)^18^ and the recommendations of Egypt's guide for the care and use of laboratory animals^19^. The current study adheres to the ARRIVE Guidelines for reporting in vivo experiments^20^. All efforts were made to curb the distress of rats during the experimental period.

### Experimental animals

The present study was conducted on 30 male Wistar rats (aged 2–3 months). Rats were obtained from the animal house of the Medical Research Institute, Alexandria University, Egypt. Animals were kept 5 per cage at 23 °C in a 12 h light/12 h dark cycle under good hygienic conditions and standard humidity with access to food and water.

#### Experimental design

The animals were classified into two groups according to diet they were receiving: 1. Group I (Control group): 15 male rats that were maintained under normal diet (13.5% kcal fat), 2. Group II (HFD group): 15 male rats that were feeding with high fat diet (60% kcal fat) (D12492; Research Diets, New Brunswick, NJ) for 12 weeks^21^.

At the end of experiment, final body weight and length were recorded to calculate the body mass index (BMI). Rats were fasted for 12 h and then sacrificed by cervical dislocation under anesthesia (ketamine 100 mg/kg and xylazine 10 mg/kg intraperitoneally)^22^. Blood samples were collected, centrifuged at 1000×*g* for 20 min at 4 °C to separate the sera for biochemical analysis.

Cartilage tissues of both groups were gently dissected, cleaned with saline, and divided into two parts. The first part was homogenized in a phosphate buffered saline (PBS) in ratio of 1:9, centrifuged at 4 °C for 10 min at 10,000×*g*, and the supernatant was collected and stored at − 80 °C for determination of resistin, interleukin-1β (IL-1β), caspase 3, MMP-13, ADAMTS 5 and ATG-5. The second part was used for RNA extraction to analyze gene expression. The third part was used for histopathological analysis.

### Biochemical analysis

Serum levels of fasting serum glucose (FBG), triglycerides (TG), total cholesterol (TC), and high density lipoprotein-cholesterol (HDL-C) were assayed using commercial available kits (Bio Med Diagnostic INC, USA). Low density lipoprotein-cholesterol (LDL-C) was estimated according to the Friedewald’s equation: LDL-C (mg/dl) = TC − (HDL-C) − (TAG/5)^23^.

The serum levels of insulin were assayed using immunoassay kit (EMD Millipore USA). The homeostasis model assessment index for insulin resistance (HOMA-IR) was then calculated using the following formula^24^:

$${\text{HOMA-IR}} = \frac{{{\text{Fasting insulin }}( {{\upmu\text{IU}}/{\text{ml}}} ) \times {\text{Fasting glucose }}( {{\text{mg}}/{\text{dl}}} )}}{22.5 \times 18}$$

### Determination of resistin, IL-1β, caspase 3, MMP-13, ADAMTS 5 and ATG-5

IL-1β and caspase 3 levels were evaluated in cartilage homogenates using ELISA kits purchased from (MyBioSource, Inc., San Diego, CA, USA and Cusabio Biotech Co., Ltd), respectively according to their manufacturer’s instructions. A competitive ELISA kit was used for the determination of serum and cartilage resistin levels (LifeSpan BioSciences, Inc.). Matrix metalloproteinase-13 (MMP-13) and ADAMTS 5 (aggrecanase-2) were measured in cartilage homogenates using Sandwich ELISA kits purchased from (Life Span Biosciences, Inc and Mybiosource), respectively. ATG-5 protein levels were also measured in cartilage using ELISA kits purchased from (Mybiosource). The total protein concentration was determined using Lowry’s method^25^.

### RNA extraction and real-time RT-PCR

30 mg of cartilage tissues were used for total RNA extraction using the miRNeasy Mini Kit (Qiagen, Germany) according to the manufacturer’s instructions and the concentration and integrity of extracted RNA were checked using nanodrop. The reverse transcription of the extracted RNA was performed using Reverse transcription (RT) was performed by TOPscript RT DryMIX kit (dT18/dN6 plus) (Enzynomics, Korea) according to the manufacturer instructions. The tissues expression of mammalian target of rapamycin (mTOR), beclin-1, light chain 3 (LC3), autophagy related 5 (ATG5), matrix metalloproteinase-9 (MMP-9), toll-like receptor 4 (TLR4) and IL-1β were quantified in the cDNA by CFX Maestro Software (Bio-Rad, USA) using QuantiNova SYBR Green PCR Kit (Qiagen, Germany). Quantitative PCR amplification conditions were adjusted as an initial denaturation at 95 °C for 10 min and then 45 cycles of PCR for amplification as follows: denaturation at 95 °C for 20 s, annealing at 55 °C for 20 s and extension at 70 °C for 15 s. The housekeeping gene 18S rRNA was used as a reference gene for normalization. The primers used for the determination of rat genes are presented in supplementary material (Table S1). The relative change in mRNA expression in samples was estimated using 2^−ΔΔCt^ method.

### Cartilage histology

The knee joints of adult rats were fixed in 10% neutral buffered formalin for 48 h. Then placed into hydrochloric acid to decalcify. After decalcification process, the knee joints were cut into approximately 2 equal halves. The joints were processed for paraffin embedding and sectioned at 5 μm for Hematoxylin and Eosin (H&E) stain for histopathological examination.

### Statistical analysis

Data were analyzed using SPSS software package version 18.0 (SPSS, Chicago, IL, USA). The data were expressed as mean ± SE and analyzed using Student independent *t* test to compare between different groups. The p value was assumed to be significant at p < 0.05^26^. The correlation coefficients (r) between different assayed parameters were evaluated using Pearson correlation coefficient; p < 0.05 was considered as the significance limit for all comparisons.

## Results

### Effect of HFD feeding on anthropometric and biochemical parameters

**Figure 1:**
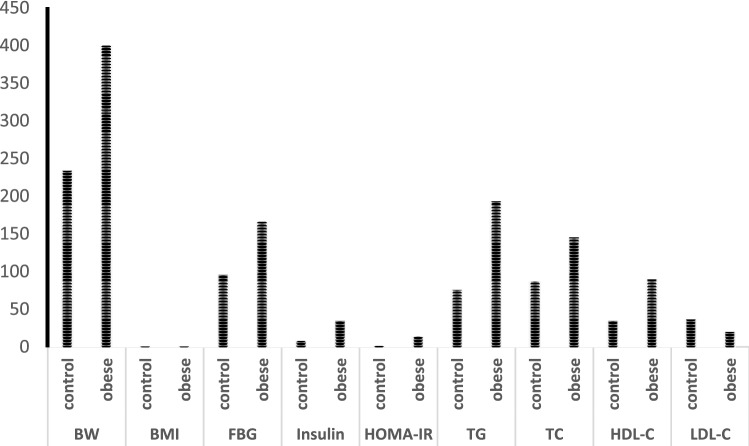
Data were illustrated as mean ± SD, *p < 0.05, indicating a statistically significant difference when compared with control group using independent sample *t* test.

As shown in Fig. 1, our results showed significant elevation in the body weight and body mass index (BMI) in obese rats compared to control by about 71, 61%, respectively. The serum levels of fasting glucose and insulin as well as HOMA-IR index were all significantly increased by about 72, 333, 652%, respectively in the same group compared to control. In addition, HFD feeding induced a significant increase in serum TG, TC and LDL-C levels by about 154, 67 and 148%, respectively compared with normal rats, whereas the serum HDL-C levels were significantly reduced by about—45%.

### Effect of HFD feeding on resistin levels and TLR4 expression

**Figure 2:**
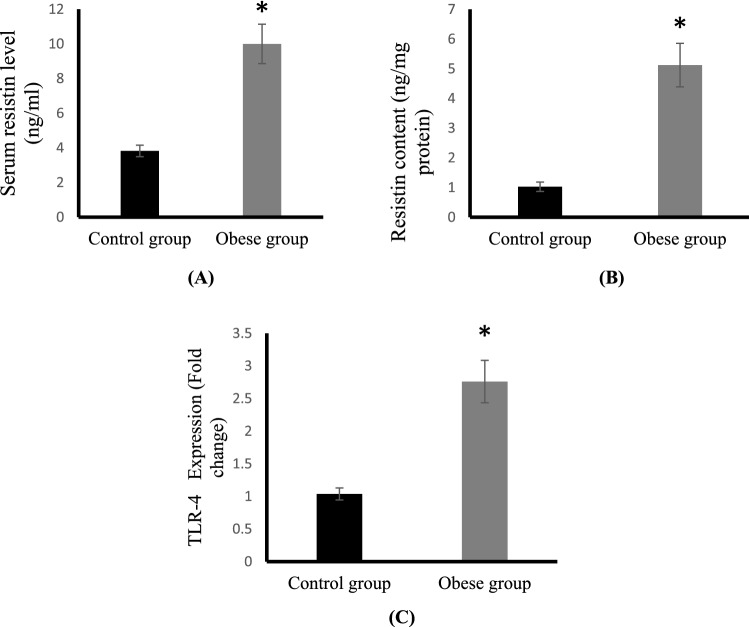
Serum resistin levels (ng/ml) (**A**), cartilage resistin content (ng/mg protein) (**B**) and cartilage TLR4 mRNA expression (**C**) in the control and obese groups.

Obese rats showed highly significant increase in the serum level and cartilage tissue content of resistin by about 161, 398%, respectively compared to control rats (Fig. 2A,B). The mRNA expression of TLR4 was significantly upregulated in cartilage of obese group compared to control (Fig. 2C).

### Effect of HFD feeding on osteoarthritis markers

**Figure 3:**
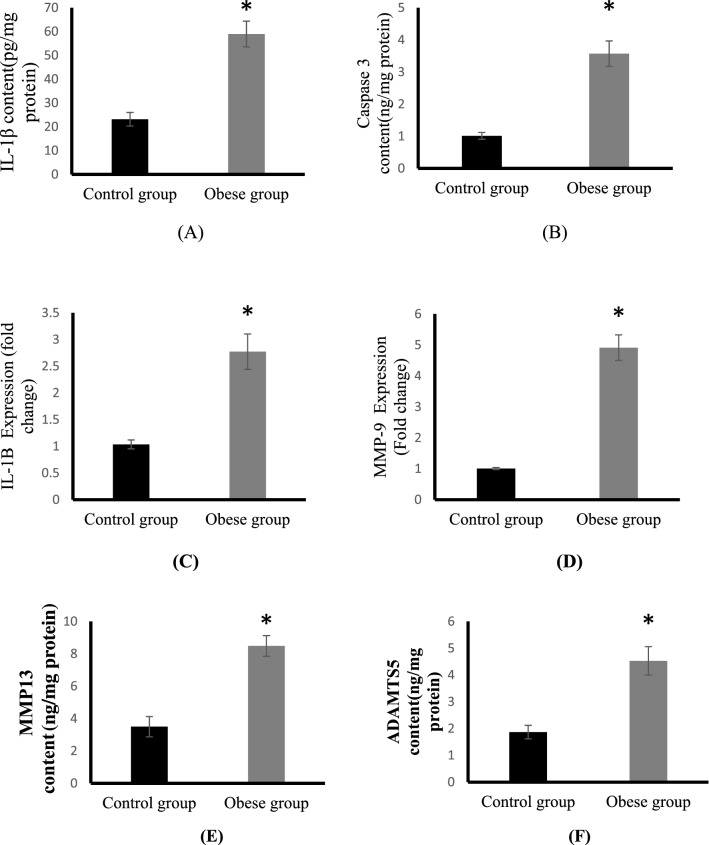
Cartilage mRNA expressions of IL-1β (pg/mg protein) (**A**) and caspase 3 (ng/mg protein) (**B**) IL-1β expression (**C**) and MMP-9 expression (**D**), MMP13 (ng/mg protein) (**E**) and ADAMTS5 (ng/mg protein) (**F**) in the control and obese groups.

As shown in Fig. 3, all osteoarthritis parameters including IL-1β, caspase 3 and MMP-9 were significantly increased in the obese group compared to control. Obese rats showed highly significant increase in cartilage contents of IL-1β and caspase 3 by about 154, 252%, respectively compared to control rats (Fig. 3A,B). The Cartilage tissue expression (fold change) of IL-1β and MMP-9 are shown in (Fig. 3C,D), respectively. Our results revealed that HFD promoted a significant upregulation of IL-1β expression by threefold in obese group compared to control, whereas MMP-9 expression was significantly increased fivefold in the same group than control group. Also, HFD caused a significant increase in MMP13 and ADAMTS5 protein levels in cartilage (Fig. 3E,F).

### Effect of HFD feeding on autophagy markers

**Figure 4:**
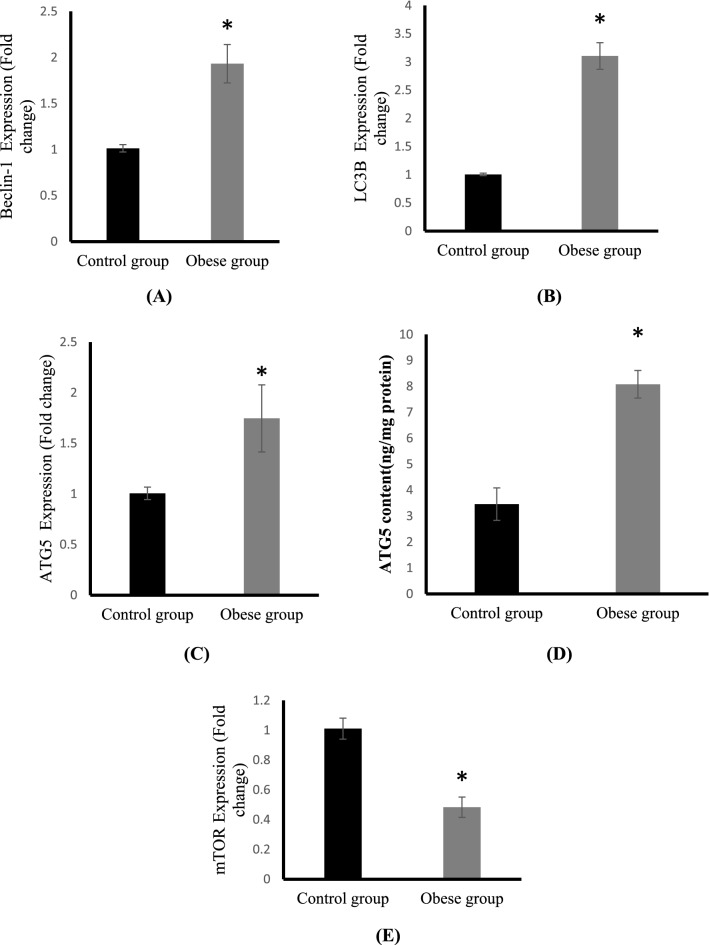
Cartilage mRNA expressions of Beclin-1 (**A**), LC3B (**B**), ATG5 (**C**), ATG5 protein levels (**D**) and mTOR expression (**E**) in the control and obese groups.

The expression of autophagy genes including Beclin-1, LC3B and ATG5 and the negative regulator of autophagy, mTOR in cartilage tissue are shown in Fig. 4. HFD feeding induced a significant upregulation of mRNA expressions of beclin-1 and LC3B in cartilage tissues by two fold as compared to control (Fig. 4A,B). A significant increase in ATG5 mRNA expression to one and half fold was also detected in the cartilage tissues of obese rats compared with cartilage tissues of control rats (Fig. 4C). In addition, a significant increase in ATG-5 protein levels were detected in the cartilage of HFD-fed rats (Fig. 4D).

As shown in Fig. 4E, compared with control group, the mRNA expression of mTOR in articular cartilage was significantly downregulated and reduced to half in the obese group.

### Correlation results

**Figure 5:**
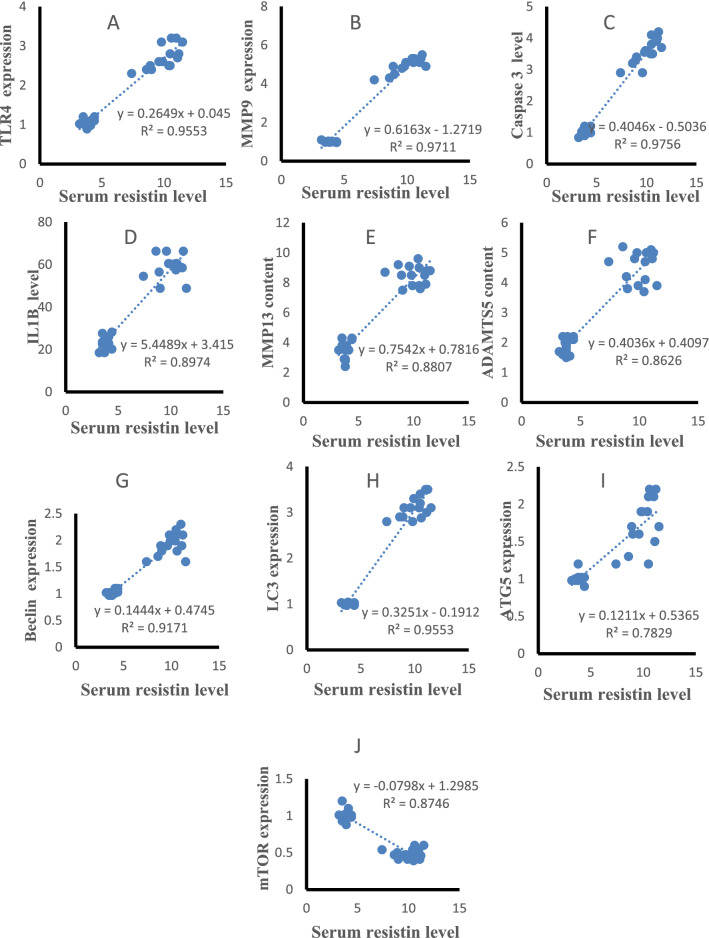
Correlations of serum resistin levels with other studied parameters in control and HFD groups.

**Figure 6:**
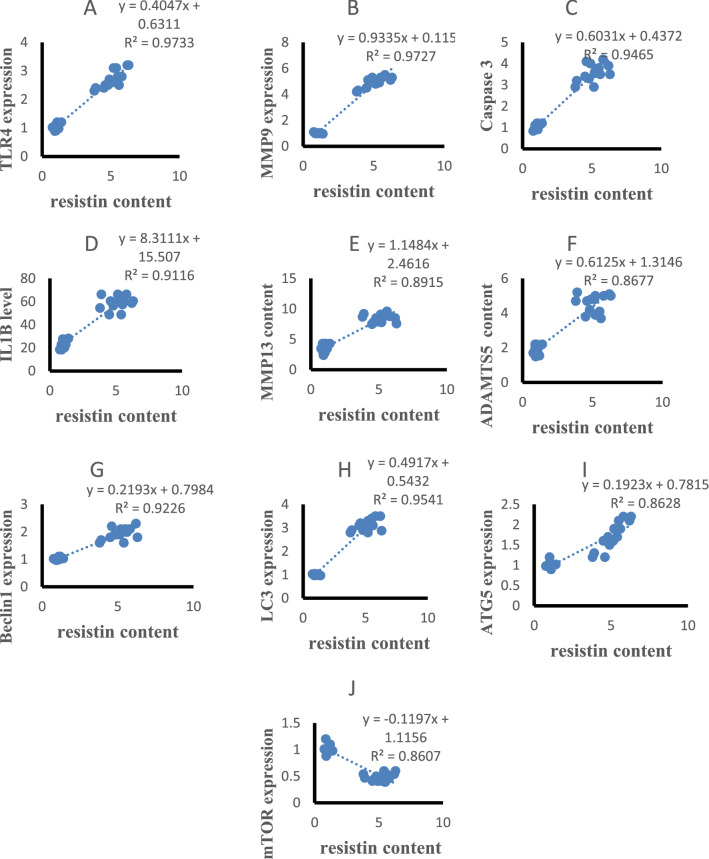
Correlations of cartilage resistin levels with other studied parameters in control and HFD groups.

**Figure 7:**
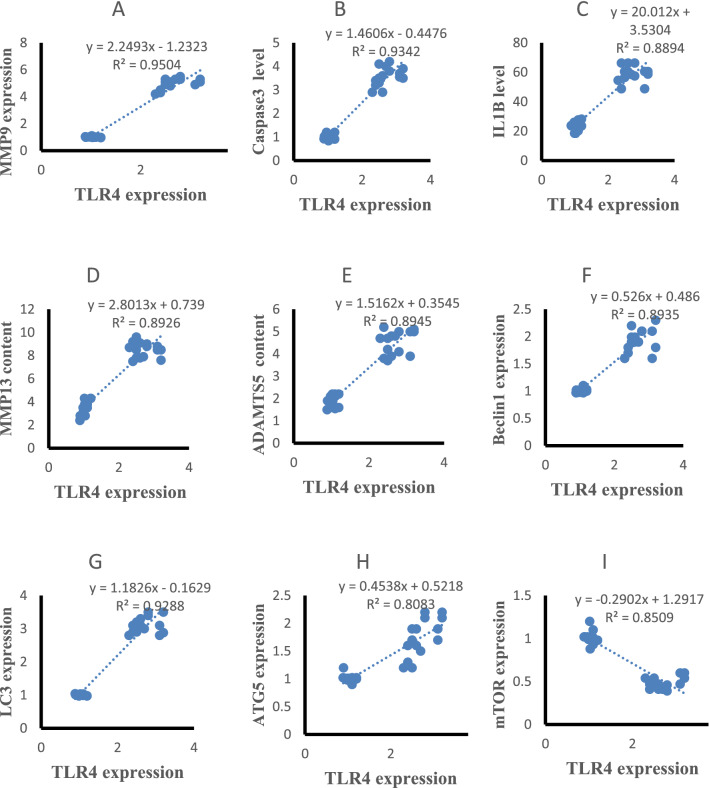
Correlations of cartilage TLR4 expression with other studied parameters in control and HFD groups.

The statistical analysis using Pearson correlation revealed significant positive correlations of serum resistin with TLR4 and MMP9 expression (r = 0.947, p < 0.001, r = 0.951, p < 0.001, Fig. 5A,B), caspase 3, IL-1β, MMP-13 and ADAMTS5 content (r = 0.915, p < 0.001, r = 0.955, p < 0.001, r = 0.951, p < 0.001, r = 0.944, p < 0.001, Fig. 5C–F), Beclin-1, LC3B, ATG5 expression (r = 0.938, p < 0.001, r = 0.948, p < 0.001, r = 0.840, p < 0.001 Fig. 5G–I) and negatively correlated with mTOR expression(r = − 0.924, p < 0.001, Fig. 5J) in all studied groups. Cartilage resistin content was positively correlated with TLR4 and MMP9 expression (r = 0.934, p < 0.001, r = 0.955, p < 0.001, Fig. 6A,B), caspase 3, IL-1β, MMP-13 and ADAMTS5 content (r = 0.955, p < 0.001, r = 0.910, p < 0.001, r = 0.962, p < 0.001, r = 0.952, p < 0.001, Fig. 6C–F), Beclin-1, LC3B, ATG5 expression (r = 0.918, p < 0.001, r = 0.949, p < 0.001, r = 0.815, p < 0.001 Fig. 6G–I) and negatively correlated with mTOR expression (r = − 0.944, p < 0.001, Fig. 6J) in all studied groups. TLR4 expression was positively correlated with MMP9 expression (r = 0.963, p < 0.001, Fig. 7A), caspase 3, IL-1β, MMP-13 and ADAMTS5 content (r = 0.946, p < 0.001, r = 0.927, p < 0.001, r = 0.967, p < 0.001, r = 0.916, p < 0.001, Fig. 7B–E), Beclin-1, LC3B, ATG5 expression (r = 0.894, p < 0.001, r = 0.962, p < 0.001, r = 0.883, p < 0.001 Fig. 7F–H) and negatively correlated with mTOR expression (r = − 0.947, p < 0.001, Fig. 7I) in all studied groups.

### Histopathological results

**Figure 8:**
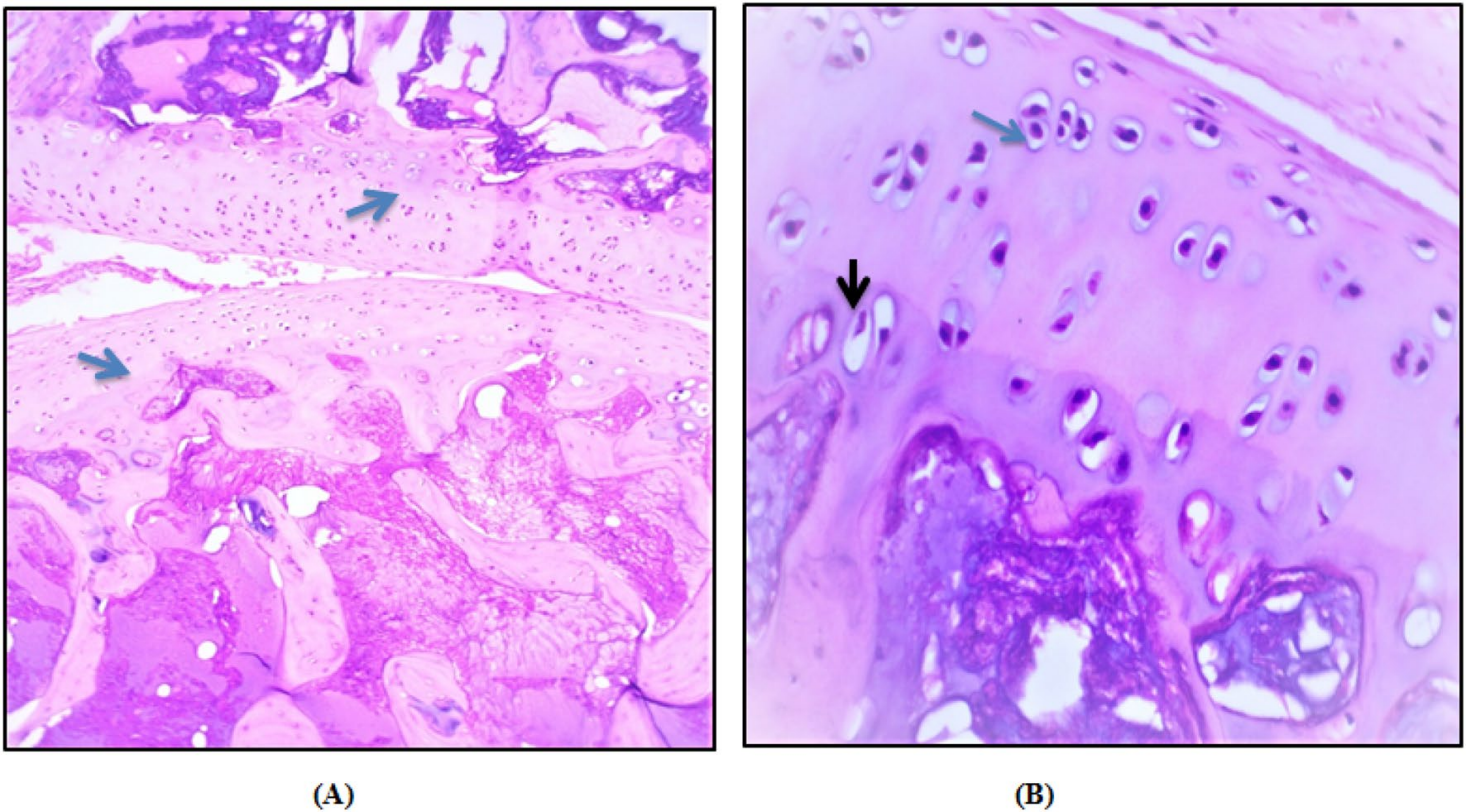
Photomicrographs illustrating the normal microscopic features of sections of articular cartilage of the adult rat. (**A**) Articular cartilage of proximal portion of the tibia the distal portion of the femur is separated into calcified and noncalcified region by a basophilic tidemark (blue arrow). Magnification × 10. (**B**) Chondrocytes lying centrally within its lacunae with centrally located nuclei (blue arrow) and hypertrophic chondrocytes (black arrow) are located in the calcified cartilage near the subchondral bone. Magnification × 40.

In the control group, H&E staining of cartilage showed a preserved morphological structure with no signs of cartilage degradation. The surface of knee cartilage appeared white, shiny, and firm. It has a smooth surface without fissures. Further, Non-calcified and calcified portions of articular cartilage were separated by a basophilic tidemark (Fig. 8A). The chondrocytes were lying centrally within its lacunae (Fig. 8B). Moreover, they showed finely granular cytoplasm and centrally located nuclei. The most hypertrophic chondrocytes was observed to locate in the calcified cartilage zone.

**Figure 9:**
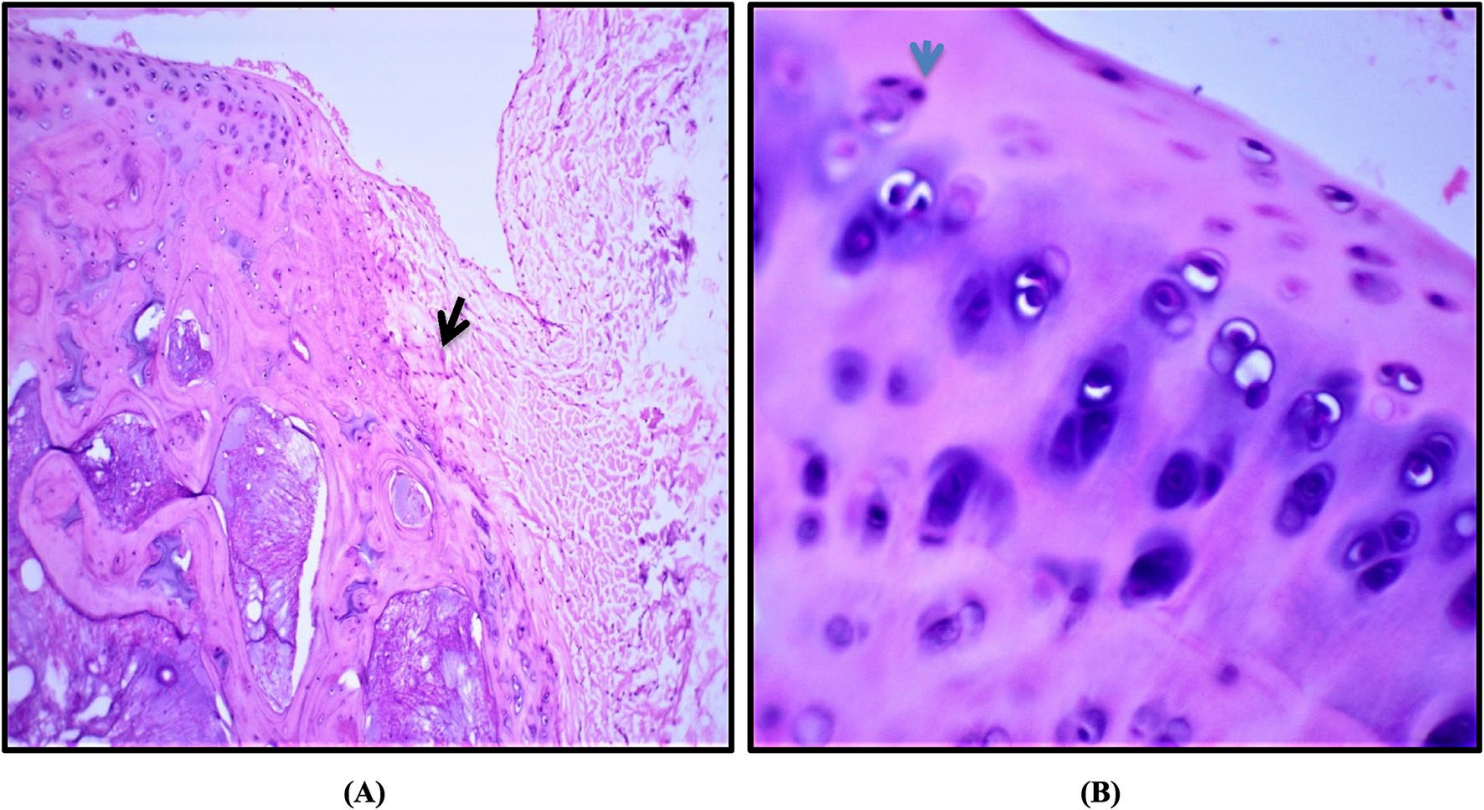
Photomicrographs illustrating the lesions resulting from HFD. (**A**) Fibrillation and fissuring of articular cartilage progressed to erosion of non-calcified cartilage was shown (black arrow) and the tidemark is no longer intact. Magnification × 10. (**B**) Most of chondrocytes were highly destructed with no clear lacunae (blue arrow) and pyknotic nuclei was also observed. Magnification × 40.

Knee cartilage of the HFD group showed dull and irregular surface with deep surface cleft. Moreover, disappearance of cells from tangential zone was observed with enlargement and disorganization of the chondrocytes in other zones which are not arranged in columns. This was accompanied by changes in the cartilaginous matrix including fibrillation at the articular surface (Fig. 9A). Also, the subchondral bone showed Fibrillation and the basophilic tidemark is no longer intact. A significant chondrocyte loss but with collagen retention was also observed (Fig. 9B). These structural alterations led to reduction of cartilage thickness of the superficial and middle zones.

## Discussion

Obesity is considered one of the well-recognized and modifiable risk factors of knee osteoarthritis (KOA)^27^. Previous studies have demonstrated that obesity causes a state of low-grade chronic inflammation that consists of inflammatory cytokines and abnormal metabolites, leading to cartilage matrix impairment, synovitis and subchondral bone remodeling^28,29^. In the present study, the protocol implemented to generate obesity was a 12-week high fat diet, which led to a significant increase in the total body mass and as expected was associated with dyslipidemia, hyperglycemia and insulin resistance. Our findings showed that short-term HFD feeding for 12 weeks induced a significant increase in mRNA expressions of matrix metalloproteinase-9 (MMP-9) and interleukin-1β (IL-1β) as well as the protein levels of IL-1β, matrix metalloproteinase-13 (MMP-13), ADAMTS 5 (aggrecanase-2) and caspase-3 in the cartilage tissue, leading to articular cartilage degeneration. This was confirmed by histopathological investigation of articular cartilage of HFD-fed rats. In the present study, H&E staining showed clear characteristic features of OA in HFD group as compared to the control group. The number of chondrocytes was markedly decreased, which could be explained by chondrocyte death due to necrosis or apoptosis as pyknotic nuclei were observed. Also, fissures gradually extended through the full thickness of the cartilage and into subchondral bone led to complete loss of cartilage. This was concordant with the previous works in knee joints used different model systems. Sun et al.^30^ found that obesogenic diets consisting of high-fat, high-carbohydrate promoted systemic and local synovial inflammation and contributed to the development of OA. In another study by Collins et al.^31^, a high-fat/high-sucrose (HFS) diet caused OA-like joint degeneration in the shoulders, knees, and hips in a rat model of OA. In a mouse model, it was also discovered that a 16-week high-fat diet introduction was linked to alterations in knee cartilage mechanical characteristics, which are thought to be an early predictor of OA development^32^.

Regardless of the fact that there is a well-established link between obesity and OA, the underlying mechanisms are still poorly understood. In recent years, the involvement of adipokines in the development of OA pathogenesis has gained increasing attention, and it is suggested to be crucial mediators linking obesity to chronic, low-grade inflammation and joint damage^33^.

Resistin, also known as ADSF (adipose tissue-specific secretory factor) or FIZZ3, (in inflammatory zone 3) is a novel adipokine that has been suggested to play an important role in obesity, insulin resistance and inflammation^34^. It is a cysteine-rich polypeptide secreted from adipocytes in rodents and humans; however, macrophages are the major source of resistin in humans^34^. Resistin has recently been revealed to have a role in the pathophysiology and development of OA^6^. Our findings demonstrated that HFD-induced obese rats had considerably greater serum and cartilage resistin levels than control rats.

Several research have looked at resistin's function in the pathophysiology of OA. Clinical investigations have found a substantial positive relationship between OA severity and levels of resistin in human OA serum, synovial fluid, and cartilage tissues, which is consistent with the current findings^35^. In patients with knee osteoarthritis, the serum levels of resistin were related to cartilage defects and bone marrow lesions^36^.

According to a recent research, it was shown that the levels of resistin in OA patients were higher than in healthy controls by Alissa et al.^27^, and they were positively linked with inflammatory markers. In articular cartilages from humans, the production of matrix degrading enzymes and inflammatory cytokines has been found to be increased by resistin^8,9^. Resistin has also been shown to rise systemically and locally following joint damage, and to have a direct influence on cartilage matrix turnover and cytokine production in vitro^37^. Inflammatory cytokines are known to trigger apoptosis in chondrocytes by releasing cytochrome *c* from mitochondria and activating the caspase gene^38^.

Resistin's inflammatory effects in OA have been documented^7,10^. In human chondrocytes, Zhao et al.^7^ suggested that resistin stimulates expression of proinflammatory cytokines and matrix-degrading enzymes via activation of p38 mitogen-activated protein kinase (p38-MAPK) and nuclear factor-κB (NF-κB) signaling pathways. Furthermore, Chen et al.^10^ demonstrated that resistin increases the expression of vascular adhesion molecule-1 (VCAM-1) on human synovial fibroblasts in OA by inhibiting miR 381 synthesis. Upregulation of VCAM-1 facilitates the adhesion of monocytes to synovial fibroblasts, leading to increased synovial inflammation.

In rats and humans, resistin contains a variety of functional receptors, and these receptors play a significant role in resistin-induced inflammation^6^. Toll-like receptor 4 (TLR4) is linked to metabolic inflammation and insulin resistance in obese people^39,40^ and OA patients^41^. Activation of TLR4 induces NF-κB signaling and proinflammatory cytokine production, which are both upregulated in OA joint tissues^42^.

Several studies reported that TLR4 acts as a functional receptor directly binding resistin and is involved in the proinflammatory effect of resistin in different tissues^39^. Li et al.^43^ found that resistin binds to TLR4 and causes macrophage infiltration by increasing CCL4 expression in the nucleus pulposus cells of the human intervertebral disc. TLR4-mediated activation of swine alveolar macrophages by resistin was shown to promote the inflammatory cytokines production^44^. Moreover, Miao et al.^45^ demonstrated that resistin suppressed autophagy in mice hypothalamus through TLR4, which subsequently led to increased inflammation. In the present study, we demonstrated that TLR4 expression is increased in cartilage tissue of the obese group compared with control. It was positively associated with resistin levels, matrix metalloproteinase-9 (MMP-9) and TLR4 expressions and protein levels of IL-1β, caspase-3, MMP-13 and ADAMTS5 content. This finding indicated that TLR4 could be a putative receptor of resistin in the articular cartilage. However, more future investigations are needed to determine whether resistin directly binds to TLR-4 in the cartilage and to uncover the roles of resistin-TLR4 pathway and other resistin receptors such as decorin, receptor tyrosine kinase-like orphan receptor 1 (ROR1), and adenylyl cyclase-associated protein 1 (CAP1) in the pathophysiology and progression of OA.

Autophagy is a lysosomal degradation pathway that is necessary for regulation of energy and nutrition as well as maintenance of energy metabolism in the body^12,46^. The autophagy-related proteins (ATG) (the core machinery) and accessory proteins are recruited in a spatially coordinated manner^13^. Among ATG proteins, autophagy related 5 (ATG5), beclin-1 and light chain 3 (LC3) are regarded the most commonly targeted genes and widely used markers for monitoring autophagy activity and flux^47,48^. Autophagy process is also controlled by the mammalian target of rapamycin (mTOR), a serine/threonine protein kinase that is a major negative regulator of autophagy^49^.

Recently, deregulation of autophagy has been implicated in the pathogenesis and progression of both obesity^50^ and OA^14,15^, but the results regarding changes in autophagy are contradictory. The current research revealed significant upregulation of ATG5, Beclin-1 and LC3 mRNA expressions and downregulation of mTOR mRNA expression as well as increased ATG5 protein levels in the cartilage of obese group, which is consistent with Shin et al.^14^, study on monosodium iodoacetate (MIA)-induced rodent model of OA. We suggested that the upregulation of autophagy in this context may be a part of a cellular defense mechanism under obesity-associated stresses.

Our results are in contrast with other reports that showed reduced autophagy level in OA cartilage^15^ and alleviated OA severity by autophagy activation^51^. With the progression of OA, mTOR was shown to rise, resulting in the repression of autophagy in articular cartilage and the encouragement of cartilage degradation^52^.

It has been shown that autophagy is upregulated in chondrocytes and cartilage during the early degenerative phase of OA to regulate changes in OA-like gene expression by managing oxidative stress and apoptosis. Thus, autophagy is an adaptive, protective response in cartilage, whereas reduced autophagy leads to cell death as cartilage degrades^53^.

In Yao et al., study^54^, HFD feeding over 28 weeks generated a reduction in beclin1 and LC3B protein expression and an increase in the expression of p-mTOR in articular cartilage, resulting in OA-like lesions, which contradicts our results. This might be linked to the length of time on HFD. Thus, we suggested that the duration of HFD consumption is one of the most important aspects affecting autophagic response in the cartilage.

In recent years, the relationship between resistin/TLR4 signaling and autophagy has been studied. Miao et al.^45^ showed that resistin/TLR4 is regarded a regulatory pathway of neuronal autophagy, but the role of resistin/TLR4 in cartilage autophagy was not yet investigated and remains unknown. Our findings revealed that serum and cartilage resistin levels as well as TLR4 expression were positively associated with cartilage expressions of ATG5, Beclin-1 and LC3 expressions and negatively correlated with mTOR, suggesting that resistin and its receptor, TLR4 may have a role in the activation of autophagy and subsequent cartilage damage. However, such an assumption will require further studies to be established and to determine if there is a direct link between resistin/TLR4 signaling and autophagy in chondrocytes.

In conclusion, this study indicated that short-term HFD increases resistin levels and upregulates autophagy-related genes in the cartilage, which may contribute to OA development. This finding provides a novel insight into the underlying mechanisms linking obesity to OA pathogenesis and opens the door for future work investigating the role of resistin/TLR4/autophagy pathway.

## Supplementary Information

Supplementary Table S1.

## Data Availability

The datasets used and/or analysed are available from the corresponding author on reasonable request.
