# Supplementary material for: Effect of short-term high fat diet on resistin levels and expression of autophagy-related genes in the cartilage of male rats
Source: Sci Rep. 2022 Sep 12;12:15313. doi: 10.1038/s41598-022-19481-1 (PMC9468003; doi:10.1038/s41598-022-19481-1)
Supplement: Supplementary file 1 — Supplementary Table S1. [file 41598_2022_19481_MOESM1_ESM.docx]

Table S1: Primers used for the PCR amplification

| **Gene** | | **primer sequence** | | |
| --- | --- | --- | --- | --- |
| **18S rRNA**  **(Reference gene)** | | **F:** | | GTAACCCGTTGAACCCCATT |
|  |  | **R:** | | CAAGCTTATGACCCGCACTT |
| **mTor** | | **F:** | | TTGGAGTGGCTGGGTGCTGA |
|  |  | **R:** | | AAGGGCTGAACTTGCTGGAA |
| **Beclin-1** | | **F:** | | TTGGCCAATAAGATGGGTCTGAA |
|  |  | **R:** | | TGTCAGGGACTCCAGATACGAGTG |
| **LC3B** | | **F:** | | CAGGATCCATGCCGTCCCAGAAGACC |
|  |  | **R:** | | GTCCCTTTTTGCCTTGGTAG |
| **ATG5** | | **F:** | | AACTGAAAGAGAAGCAGAACCA |
|  |  | **R:** | | TGTCTCATAACCTTCTGAAAGTGC |
| **MMP-9** | **F:** | | AGGTGCCTCGGATGGTTATCG | |
|  | **R:** | | TGCTTGCCCAGGAAGACGAA | |
| **TLR-4** | **F:** | | CGC TTT CAC CTC TGC CTT CAC TAC AG | |
|  | **R:** | | ACA CTA CCA CAA TAA CCT TCC GGC TC | |
| **IL-1β** | **F:** | | CACCTTCTTTTCCTTCATCTTTG | |
|  | **R:** | | GTCGTTGCTTGTCTCTCCTTGTA | |
